# CSI NGS Portal: An Online Platform for Automated NGS Data Analysis and Sharing

**DOI:** 10.3390/ijms21113828

**Published:** 2020-05-28

**Authors:** Omer An, Kar-Tong Tan, Ying Li, Jia Li, Chan-Shuo Wu, Bin Zhang, Leilei Chen, Henry Yang

**Affiliations:** 1 Cancer Science Institute of Singapore, National University of Singapore, Singapore 117599, Singapore; kttan@nus.edu.sg (K.-T.T.); csiliyi@nus.edu.sg (Y.L.); csilijia@nus.edu.sg (J.L.); csiwc@nus.edu.sg (C.-S.W.); csizha@nus.edu.sg (B.Z.); polly_chen@nus.edu.sg (L.C.); 2 Department of Anatomy, Yong Loo Lin School of Medicine, National University of Singapore, Singapore 117594, Singapore

**Keywords:** NGS data analysis, bioinformatics pipelines, NGS pipelines

## Abstract

Next-generation sequencing (NGS) has been a widely-used technology in biomedical research for understanding the role of molecular genetics of cells in health and disease. A variety of computational tools have been developed to analyse the vastly growing NGS data, which often require bioinformatics skills, tedious work and a significant amount of time. To facilitate data processing steps minding the gap between biologists and bioinformaticians, we developed CSI NGS Portal, an online platform which gathers established bioinformatics pipelines to provide fully automated NGS data analysis and sharing in a user-friendly website. The portal currently provides 16 standard pipelines for analysing data from DNA, RNA, smallRNA, ChIP, RIP, 4C, SHAPE, circRNA, eCLIP, Bisulfite and scRNA sequencing, and is flexible to expand with new pipelines. The users can upload raw data in FASTQ format and submit jobs in a few clicks, and the results will be self-accessible via the portal to view/download/share in real-time. The output can be readily used as the final report or as input for other tools depending on the pipeline. Overall, CSI NGS Portal helps researchers rapidly analyse their NGS data and share results with colleagues without the aid of a bioinformatician. The portal is freely available at: https://csibioinfo.nus.edu.sg/csingsportal.

## 1. Introduction

Next-generation sequencing (NGS) has become a routine in biomedical research thanks to its proven significance and rapidly decreasing cost. Today, an overwhelming number of sequencing protocols are available by various providers, and more of them are to be developed in the near future as the underlying technology advances. In parallel, bioinformatics tools and packages to analyse the growing NGS data are also expanding, at the expense of increasing redundancy, technicality and complexity, which often alienates the wet lab biologists from understanding the data that they have generated. On the other hand, emerging technologies such as supercomputers (e.g., National Supercomputing Centre Singapore, NSCC, https://www.nscc.sg/) and cloud computing (e.g., Amazon Web Services, AWS, https://aws.amazon.com/) offer large-scale parallel computations with high speed, memory and storage, to efficiently deal with the big data generated by the NGS platforms. These technologies, however, are still offering high-cost services, which may sometimes even exceed the cost of the sequencing itself. However, these options do not eliminate the necessity for a local bioinformatician to perform the downstream analysis and to interpret the results—unless paid for additional bioinformatics analysis service—whose task is to render the computer-generated data to the biological knowledge to address the research questions in query. Despite these facts, surprisingly, there are only a handful of attempts to build up comprehensive NGS data analysis platforms utilising the available tools and the existing technologies for the benefit of the community at free of charge (Galaxy [1] https://usegalaxy.org/, Maser [2] https://cell-innovation.nig.ac.jp/maser/).

Addressing these issues, in order to facilitate NGS data analysis and sharing, aiming to bridge the gap between biologists and bioinformaticians, we have developed CSI NGS Portal as a freely accessible, easy-to-use and comprehensive online platform, offering well-established and fully automated bioinformatics pipelines at the service of the community. Currently, the portal covers more than 10 frequently used NGS data types, committed to expand, and offers one-click data analysis and sharing. A simple and intuitive interface with tabular structure across the website greatly enhances user experience by keeping the data well-organised, easily accessible and queryable. The portal has proven to be successful and useful during its internal uptime in the last three years, commencing to extend its scope to the globe.

## 2. Results

### 2.1. Website Framework

**Figure 1:**
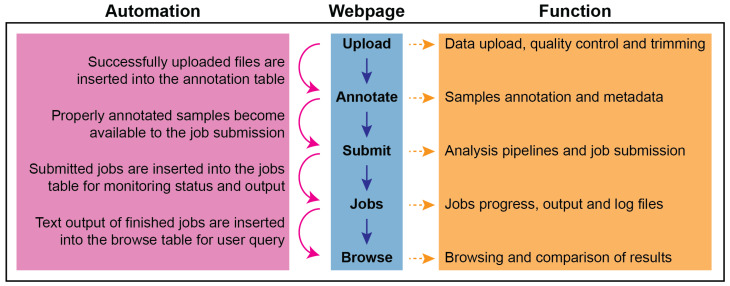
Website Framework of CSI NGS Portal. The middle panel shows the logical flow of the website from top to the bottom, whereas the automated steps of the data transitions from one page to another are described in the left panel, and the function of each page expecting user input/action is given in the right panel. 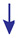: Logical flow; 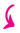: Automated step; 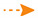: Page function.

The website framework of CSI NGS Portal consists of five major steps (“Upload”, “Annotate”, “Submit”, “Jobs”, “Browse”), each of which is built as an individual webpage (Figure 1). Although each page is independently accessible on the website menu, they are interconnected via automated data transitions, i.e., the output of each step is reflected as the input of the next step. More specifically, successfully uploaded files via the “Upload” page are inserted into the annotation table on the “Annotate” page, and properly annotated samples become available to the job submission on the “Submit” page, and status/progress of the submitted jobs can be monitored on the “Jobs” page, and, finally, text output of the finished jobs can be queried at the “Browse” page. All the pages with a table structure (“Annotate”, “Jobs”, “Browse” pages) are equipped with advanced features (search, filter, sort, edit, export and share options) to enhance user experience and data organisation. Each page has a “README” section to provide a guide about the usage and the expected input/output data, and explained further as follows:

### 2.2. Upload

**Figure 2:**
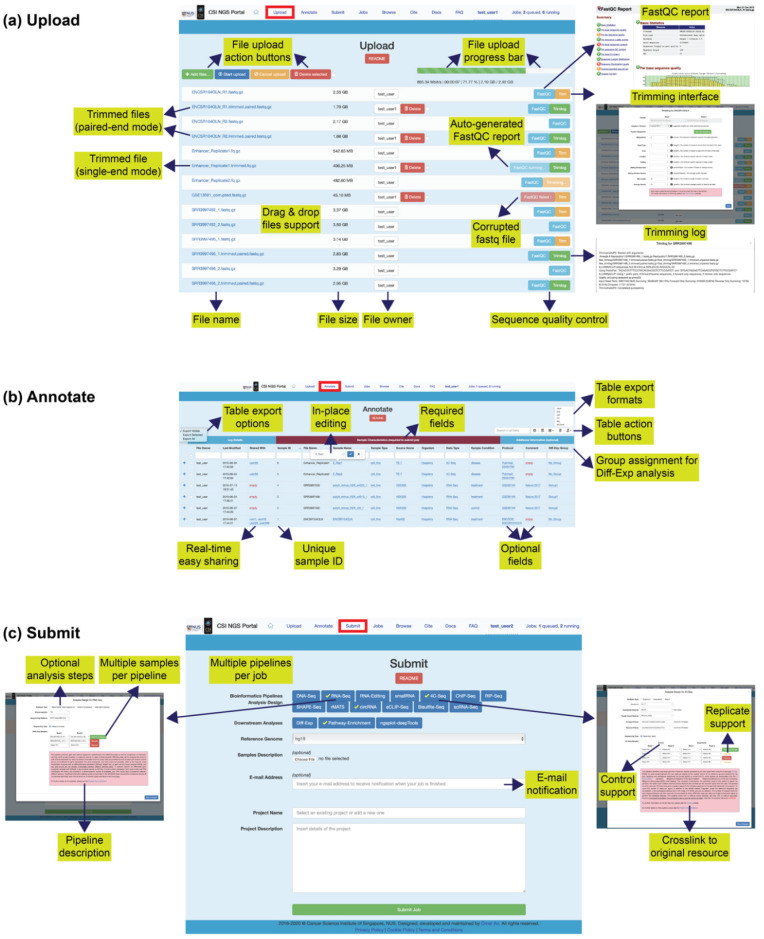

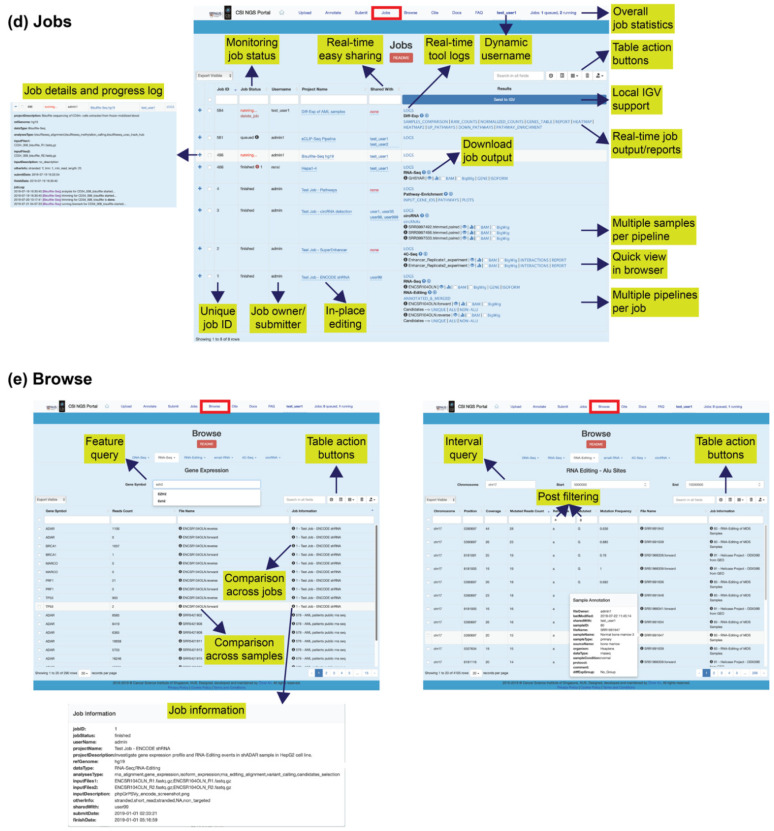
Website Interface and Usage of CSI NGS Portal. (**a**) Upload page, (**b**) Annotation page, (**c**) Submit page, (**d**) Jobs page, and (**e**) Browse page. Key features and usage information are highlighted in text boxes. Further details on the quotas, rules and usage instructions are given on the “README” sections on the website.

This page (Figure 2a) allows for uploading raw data files in the FASTQ [3] format (http://maq.sourceforge.net/fastq.shtml). All the pipelines on the portal start from the FASTQ file, followed by genome/transcriptome alignment, in order to standardise the data processing with a suitable mapper for the specific task and refrain user from the tedious alignment step. The file format requirements and the restrictions are given on the website. Successfully uploaded files are displayed as downloadable links including full file name, file size, file owner and action buttons for sequence quality check and processing, whereas failed uploads display an appropriate error message. A FastQC (a quality control tool for high throughput sequence data, https://www.bioinformatics.babraham.ac.uk/projects/fastqc/) report for each file is auto-generated in the background upon completion of the upload, allowing users to check the sequencing quality via a set of quantitative and visual metrics. A failed FastQC run is also displayed, indicating that the FASTQ file is corrupted, and such a file should not be proceeded to submit a job. In this case, user is expected to delete the corrupted file, fix the issue and re-upload the corrected file until the FastQC report is successfully generated. If needed, a trimming interface is also available employing Trimmomatic [4] to trim the adapter/primer/barcode or other custom sequences and remove the low-quality reads with a variety of options. Dragging and dropping of multiple files/folders are supported for the file upload interface, and batch upload/cancel/delete of files are available with a single click. A progress bar on top of the page displays the overall upload status showing upload speed, estimated time remaining, upload percentage and upload size out of overall size. For fair usage, there are quota restrictions on the file number and the file size per user. Uploaded files may be automatically renamed to comply with the file naming rules, e.g., spaces are replaced with underscores. Closing browser tab or internet disconnection will interrupt active file uploads. The uploaded files are private to the user and may be deleted only by the file owner or upon data expiry, whichever is earlier. Further instructions are given in the “README” section to avoid any possible problems in the next steps.

### 2.3. Annotate

Successfully uploaded files are auto-inserted into the annotation table on this page (Figure 2b) awaiting user action. A single entry is inserted per sample based on the filename regardless whether the sample comes from single-end (1 file) or paired-end (2 files) library, provided that the filenames follow the naming rules explained on the website. An incremental, unique and stable id is assigned to each sample, in addition to the unique filenames and custom sample names. The sample annotation consists of “required” and “optional” sections with predefined fields, where the former must be fully filled before job submission as it contains relevant information to the pipeline. All the editable fields offer “in-place” editing, i.e., editing by clicking directly on the html element rather than using a separate panel or dialog box and without reloading the page, which makes sample annotation a quick and easy task. The annotation may be modified and shared only by the file owner, i.e., user who has uploaded the files, and remains visible as long as the raw file exists on the “Upload” page. For circRNA/smallRNA/RNA-Seq samples, “Diff-Exp Group” must be specified to perform a “Diff-Exp” analysis to determine the sample groups to be compared (“contrast” parameter in DESeq2 [5], explained further in the Supplementary Data). The sample annotations are permanently kept in the database, and restored upon re-uploading of the same data with identical filenames, saving time for the users who need to re-analyse the same samples in the future.

### 2.4. Submit

This page (Figure 2c) allows job submission to a comprehensive number of bioinformatics pipelines for NGS data analysis (Table 1). Each pipeline has a user-friendly modal window with a simple interface for setting up the analysis design for the job. The required inputs may vary among the pipelines, although certain inputs such as sample files are common to all major pipelines. Wherever needed, information boxes are available to explain the pipeline options and parameters in further detail, as well as cross links to help direct users to the original documentation of the integrated tool. A number of control functions are also implemented at the back-end to prevent possible user mistakes prior to job submission. Even though the alignment step is required for all the pipelines starting from FASTQ file, the user may choose to opt out/in for certain analysis steps simply by un/selecting the check box, e.g., to skip/perform alternative splicing under RNA-Seq pipeline. Opting out for analyses not needed will benefit to the users by reducing the overall runtime for the job as well as the use of the server resources. The interface allows for adding multiple samples per pipeline, and multiple pipelines per job within a single submission, which will run in parallel as needed. Currently, hg19 is the default human reference genome available to all pipelines, and other reference genomes (hg38, mm10, mm9) are additionally available to certain pipelines. A description file such as datasheet or metadata can be optionally attached to the job submission for future reference, which can be in any format. In addition, an optional e-mail address field is available to receive notification upon job completion. Finally, a name and description of the project must be inserted before job submission for reference. The accuracy of the user inputs on this page is crucial to ensure the pipelines to run without failure, which can be further checked in the job details explained in the next section.

### 2.5. Jobs

This is the main page (Figure 2d) of the portal where the users have full control over their jobs. Specifically, users can:

(a) check submitted job details to make sure everything is correct,
(b) delete the entire job or the individual samples anytime,
(c) monitor the job status if it is queued, running or finished,
(d) monitor the job progress via real-time log with timestamp,
(e) access the output files in real-time for view/download,
(f) share/unshare job results with other users anytime.

The jobs table by default displays only the most important fields due to the space constraint, which can be expanded by selecting more columns in the “Columns” action button. Alternatively, all the fields can be viewed with the “Details” button (**+** sign) including overall job progress log. Importantly, the number of days to expiry is indicated in the job status column, after which the job and the associated data are automatically removed. The jobs can be shared with other users anytime, by simply inserting their usernames into the “Shared With” field separated by space(s) and/or comma: e.g., *topuser1, sevgi55 mike_86, john*. Likewise, usernames can be removed to unshare the job. Finally, the results column displays the output of the pipelines, as being the most important part of the page and possibly of the portal. The results become available stepwise in real-time, e.g., as the job keeps running before full completion, and all of the output is available to directly view on the browser or to download to the local computer with single click. Alignment data (in .bam, .bigwig, .vcf formats) can be directly viewed in Integrative Genomics Viewer (IGV) [37] installed on the user’s local computer without downloading the original files, allowing comparison of samples between different pipelines and even different jobs. Similarly, UCSC track hubs [38] are provided for supported pipelines to visualise and compare the job output across samples. Due to the high volume of the NGS data, users are strongly encouraged to download the output files and delete the job as soon as possible, which will help with efficient usage of the storage and faster browsing experience on the portal for everyone.

### 2.6. Browse

This page (Figure 2e) provides interactive tables to browse the job results for the pipelines with reasonable size of text output (“DNA-Seq”, “RNA-Seq”, “RNA-Editing”, “smallRNA”, “4C-Seq”, ”ChIP-Seq”, “RIP-Seq”, “circRNA”, “eCLIP-Seq”). As soon as such a job is finished, the results are automatically inserted into the database as a part of the pipeline, so that they become available for browsing and comparison. The results can be queried by either genomic feature (e.g., gene symbol, smallRNA identifier, etc.) or genomic interval (e.g., chromosome, start, end) or both depending on the pipelines used. The query returns matched results from all the jobs under the same category belonging to the user as well as those that are shared with the user. For example, assuming a user has three DNA-Seq jobs and two other DNA-Seq jobs shared with him by his colleague, he will be able to compare the mutations in *gene X* across all the samples under the five jobs in a single query. The query results can be further tuned by using the action buttons on the table.

### 2.7. Portal Features

Among many features of the portal (Table 2), the most powerful of them are its usability, modularity and flexibility. It is usable because of its simple design yet powerful functionality, and automation of not only the pipelines but also the data transitions between the pages. It is modular so that a new pipeline can be readily integrated to the portal complying with the existing website framework, providing high scalability. It is flexible so that there is no restriction for the script language used in the background or the pipeline parameters collected from the user, making virtually any tool that is compatible with a Linux environment also work on the portal.

### 2.8. Comparison to Similar Platforms

CSI NGS Portal stands as a unique platform for fully automated analysis of NGS data. It aims to be a useful resource for the researchers offering an online service for a comprehensive coverage of NGS data and pipelines. There are few other resources, however, performing a similar job in providing NGS data analysis online which are compared to CSI NGS Portal next (Table 3).

Galaxy [1] (https://usegalaxy.org/) is currently the most popular project among these resources, standing as a freely available web server and open-source software for NGS data analysis, which expanded over 10 years as a scientific workflow management system towards data intensive biomedical research. However, Galaxy is primarily designed for users who have knowledge on how to build up a bioinformatics analysis pipeline (workflow) as it provides a stepwise (or tool-based) usage rather than complete pipelines, sometimes with redundant options for the individual steps and exhaustive parameters for each step. Therefore, it may not be user-friendly for a pure biologist without any prior bioinformatics skills. In comparison, CSI NGS Portal requires minimal user inputs, assuming no advanced bioinformatics knowledge from the users, who are focused on the results rather than the procedure, thereby targeting a wider user profile standing as a real user-friendly public tool for NGS data analysis. Specifically, the actions required from the users to submit a job are (1) upload raw data, (2) annotate samples, and (3) design analysis, and each of them can be easily done with a few clicks. After the job submission, monitoring its progress, viewing/downloading/comparing the results and sharing them with other users are available in real-time on the portal. In addition, the portal provides detailed documentation of the pipelines and help on interface usage, and flexible to expand with new and customised pipelines from other labs in the future. The existing Galaxy users can still manually export the job results from CSI NGS Portal to Galaxy in supported formats (.bam, .bigwig, .vcf, .bed, .txt) for further analysis. Thus, CSI NGS Portal adds a new asset to collaboratively support biomedical research with the existing platforms.

Another online platform developed for NGS big data analysis and sharing is Maser [2] (https://cell-innovation.nig.ac.jp/maser/), offering built-in bioinformatics pipelines and genome browser for data visualisation. Although the features of the two platforms are comparable in overall, compared to Maser, CSI NGS Portal covers two times more NGS data types, committed to expand offering better scalability, require no signup allowing quicker access for the users, and provides a more user-friendly interface with non-redundant pipelines and simpler design. On the other hand, CSI NGS Portal provides UCSC track hubs [38] rather than an embedded browser for the supported pipelines (RNA-Editing, ChIP-Seq, RIP-Seq, eCLIP-Seq and Bisulfite-Seq).

It is noteworthy to mention many other efforts to analyse growing NGS data, which have been serving to the community as useful resources for years. However, these tools are not directly comparable to CSI NGS Portal as they either present pre-analysed datasets (databases), focus on a single domain (e.g., functional genomics by GenePattern [39], ZENBU [40], RNA-Seq by RAP [41], miRNA analysis by miRMaster [42]), or offer distributed bioinformatics software/framework/workflow systems consuming user’s owned resources, rather than a publicly accessible online service, which require bioinformatics capability for the installation and large resources for the usage (GobyWeb [43], Eoulsan [44], Sequanix [45], Taverna [46], Arvados (https://doc.arvados.org/), Anduril [47], BioQueue [48], and DolphinNext [49]).

## 3. Discussion

Despite the expanding applications of automated systems in the global context, not every system can be automated in perfection without human intervention; this is also true for the bioinformatics systems. Automation on CSI NGS Portal inherently bears a certain level of trade-off between speed and complexity in the NGS data analysis. For the sake of speed and ease of use, the portal is designed as to require minimal user input without advanced options, which may not always provide the power of fine-tuning the analysis for the individual tools and functions in the pipelines. However, for the majority of cases, standard pipelines with default parameters result in the desired outputs by the user and are sufficient for the downstream analyses, particularly in comparison studies.

The portal will be expanded with new NGS data analysis pipelines such as differential RNA editing analysis and neoepitope prediction for personalised medicine in the near future. Other popular pipelines will be added to the portal on demand. We also encourage the users to submit their own pipelines to share with the community. There is also room for improvement of the existing pipelines, e.g., cross-pipelines analysis, availability of more reference genomes, as well as of the website interface, e.g., built-in visualisation tool. Building a docker container for the local installation of the web application is in progress.

## 4. Materials and Methods

### 4.1. Portal Implementation

The portal has been developed in a Linux environment by using a mix of several programming languages and employing a vast number of bioinformatics tools and packages (Supplementary Table S1). Specifically, the website has been built in PHP v7 and HTML5, and the dynamic features have been implemented in JavaScript. The website runs on an Apache v2.0 server under a dedicated Linux machine (128 CPUs, 256 GB RAM, 60 TB storage) with Ubuntu v16.04.6 installation with an integrated database built with MariaDB v10. The majority of the bioinformatics tools and packages as well as their dependencies are maintained in a Conda environment (https://anaconda.com), and all the software behind the portal are regularly updated to the latest stable versions available. The website interface mainly utilises Bootstrap (https://getbootstrap.com/), a popular front-end component library and open source toolkit for developing with HTML, CSS, and JS. The user experience has been greatly enhanced by the interactive tables with search, filter, sort, edit, export, share and other functionalities owing to the Bootstrap plugins, as well as by displaying the web pages properly on different devices and platforms owing to the Bootstrap’s responsive design feature.

The bioinformatics pipelines (Table 1, Supplementary Data) consist of in-house generated scripts (written in Bash, Perl, R) and/or integrated tools mostly from freely available open source projects (written in various programming languages), and all of the pipelines have been integrated into the portal with wrapper Bash scripts. The pipelines are implemented in a modular structure, which makes it easy to add new ones in the future. To submit a job, the required inputs from the users are raw FASTQ files, basic sample annotations and simple analysis design. The job submission is subject to a queue system, which handles the jobs at the background by managing the available resources on the server. In order to utilise the resources efficiently and to obtain the results rapidly, the jobs are parallelised via multi-threading per sample wherever supported, and via simultaneous run of multiple samples per job wherever applicable.

To monitor overall job progress and to debug in case of failure, a job log has been implemented. The job log displays real-time information for each step of the pipeline with timestamp, which makes it easier to identify the source of the error upon failure. The keyword “ERROR” highlighted in red is reserved to denote the failure for this purpose. It is possible for the pipeline to continue running for the next steps even though a step has failed if they are independent from each other. Moreover, log files of the individual steps are written to a dedicated “LOGS” folder.

The portal has been designed in mind with a minimalist approach for the user input and exhaustive approach for the pipelines run. To achieve this, the processes are automated as much as possible allowing users to focus on the analysis results rather than the procedure. Provided that the user inputs have no error (we implemented functions at the backend to prevent common user mistakes), the pipelines are guaranteed to run successfully to output the expected results. In case of unexpected failure due to technical reasons such as syntax change upon software update, the job can be easily rerun by the admin with the same parameters after fixing the problem, for which no action is required from the user.

### 4.2. Website Usage

The website is publicly accessible and fully functional via major web browsers. The portal has no signup or login requirement; hence, it is open to all without any authentication; however, authorisation to data is provided via a browser cookie. Upon access to the website on the browser, a random cookie is created on the user’s local computer and associated with a stable user id and a generic username, where the latter can be changed to a personal one any time by the user, which is solely used for data sharing among users. The returning users are recognised via the browser cookie, which allows user-specific data to be available until it expires (10 days after the job finish date and 30 days since the last access to the website). Upon expiry, the user accounts and the associated data are removed from the server to restore disk space, except the data annotation, which is permanently stored in the database to avoid re-annotating the same data upon re-uploading in the future, provided that the filename is identical. For safety reasons, the cookie is computer and browser specific, which means that the users can access their data next time only by using the same browser from the same computer; otherwise, they are regarded as new users. Alternatively, for the sample annotation and the job results, the users may still access to their data on a different computer/browser by sharing them with themselves under a different username by using the “Shared With” field. For example, a user may upload data and run jobs from his/her computer at work with the username “user86_work”, and he/she can access the results from his/her computer at home sharing them with his other username “user86_home”. However, to delete or to modify the data, one still needs to use the original account “user86_work” from which the data are uploaded, or the data will be automatically removed after account expiry. Such a system ensures data privacy while keeping sharing results easy and simple. In addition to the data analysis, the portal also contains detailed documentation of the pipelines (“Docs” page) and answers to the frequently asked questions (“FAQ” page).

## 5. Maintenance

All the tools and packages used in the pipelines are regularly updated to the latest stable versions available by using a Conda environment which ensures that there are no compatibility issues. The modified or new pipelines are carefully tested before release for public. Similarly, the data sources such as annotation databases are also updated and kept in the most comprehensive version available as a rule of thumb. Nevertheless, the users are encouraged to report any bugs or make suggestions to facilitate user experience via given contact e-mail on the portal.

In addition, the website is constantly monitored for usage in order to:

(a) enhance user experience,
(b) improve portal performance,
(c) reduce common user mistakes,
(d) fix potential bugs,
(e) prevent abuse.

## 6. User Privacy and Data Security

The portal ensures user privacy and data security by taking a set of precautions on the website and the server including:

- No record of real user information, e.g., no signup or password requirement, usage of dynamic usernames for data sharing, optional e-mail address used only for job notification,
- Cryptographically secure and randomly generated cookies for user recognition and data authorisation,
- Encrypted internet connection via https protocol,
- Server protection by a strict firewall,
- User-restricted data access and full control upon sharing, i.e., unshare and delete,
- Restriction of sensitive functions to data owner, such as delete, edit, and share,
- Back-end control functions to prevent potential user mistakes,
- Backup of non-physical data i.e., sample annotations,
- Constant monitoring of website usage to prevent abuse.

To further strengthen data security, users are encouraged to pay attention to additional points including but not limited to:

- Avoid leaving computer unattended to prevent cookie theft,
- Download the results as soon as the job is finished and delete from the website,
- Share data with trusted people and with caution, e.g., a simple typo may cause sharing data with another user not intended,
- Report bugs as soon as encountered.

## 7. Conclusions

CSI NGS Portal is a unique platform which provides a free online service to researchers for fully automated NGS data analysis from raw data to final output with minimal user intervention. The portal covers most of the popular NGS data types and allows for sharing the results with colleagues easily. The website has a simple and user-friendly interface primarily designed for non-bioinformaticians, with detailed documentation of the pipelines and “README” information on the usage. With its comprehensive coverage and expanding potential, CSI NGS Portal stands as a promising and long-lasting resource fostering biomedical research.

## Figures and Tables

**Table 1:** Bioinformatics pipelines implemented on CSI NGS Portal.

Bioinformatics Pipeline	Analysis Steps	Tools and Packages	Sequencing Types	Normal/Control/Reference Samples	Replicate Samples ^a^	Overall Runtime
**1. DNA-Seq**	Genome alignment	BWA (mem) [6]	Single/Paired end	Optional ^b^	NA	~1 day
	Mutation calling	GATK4 Mutect2 [7,8]				
	Mutation annotation	ANNOVAR [9]				
**2. RNA-Seq**	Genome alignment	STAR [10]	Single/Paired end	NA	NA	~2 h
	Gene expression	HTSeq-count [11]				
	Isoform expression	Salmon [12]				
	Alternative splicing	in-house Perl				
**3. Diff-Exp**	Genes table	Bioconductor DESeq2 [5]	Single/Paired end ^c^	Required	Required (min 2 samples)	~10 min
	Genes report	Bioconductor regionReport [13]				
	Heatmap	Superheat [14]				
	Volcano	ggplot2 (Wickham 2016)				
	Pathway enrichment	Bioconductor ReactomePA [15]				
	Gene set enrichment analysis	GSEA [16]				
	Isoforms report	Bioconductor DEXSeq [17]				
**4. Pathway-Enrichment**	Enrichment plots	Bioconductor ReactomePA [15], enrichplot [18]	NA	NA	NA	~1 min
**5. RNA-Editing**	Genome alignment	BWA (mem) [6]	Single/Paired end	NA	NA	~7 h
	Variant calling	Samtools mpileup [19]				
	Candidates selection	adapted from [20]				
	AEI calculation	RNAEditingIndexer [21]				
	UCSC track hub	in-house Bash				
**6. smallRNA**	Genome alignment	NovoAlign	Single/Paired end	NA	NA	~1 h
	smallRNA expression	in-house Perl				
**7. 4C-Seq**	Genome alignment	BWA (mem) [6]	Single/Paired end	Optional	Optional (2 samples)	~10 min
	Interactions	Bioconductor r3Cseq [22]				
	Report	Bioconductor r3Cseq [22]				
**8. ChIP-Seq**	Genome alignment	Bowtie2 [23]	Single/Paired end	Required	NA	~2 h
	Peak calling	MACS2 [24]				
	Motif enrichment	HOMER [25]				
	UCSC track hub	in-house Bash				
**9. RIP-Seq**	Genome alignment	STAR [10]	Paired end	Required	Optional (2–10 samples)	~8 h
	Peak calling	in-house Bash				
	UCSC track hub	in-house Bash				
**10. SHAPE-Seq**	Transcriptome alignment	Bowtie2 [23]	Single/Paired end	Required	NA	~10 h
	Reactivity calculation	icSHAPE [26]				
	Structure prediction	RNAfold [27,28]				
**11. rMATS**	Genome alignment	STAR [10]	Single/Paired end	Required	Required(2–10 samples)	~2 h
	Alternative splicing	rMATS [29]				
**12. circRNA**	Genome alignment	STAR [10]	Single/Paired end	NA	NA	~1 h
	circRNA expression	in-house Perl				
**13. eCLIP-Seq**	Demultiplexing	eclipdemux [30,31]	Single/Paired end	Required	NA	~1 day
	Mapping	STAR [10]				
	Peak calling	clipper [32]				
	Peak normalisation	eCLIP [30,31]				
	Peak annotation	HOMER [25]				
	Motif enrichment	HOMER [25]				
	UCSC track hub	in-house Bash				
**14. Bisulfite-Seq**	Genome alignment	bowtie2 [23]	Single/Paired end	NA	NA	~3 days
	Methylation calling	Bismark [33]				
	UCSC track hub	in-house Bash				
	DMRs	metilene [34]				
**15. scRNA-Seq**	Genome alignment	STAR [10]	Paired end	NA	NA	~4 h
	Single cell analysis	Cell Ranger (10× Genomics)				
**16. ngsplot-deepTools**	Genome alignment	STAR [10], Bowtie2 [23]	Single/Paired end	NA	NA	~4 h
	Plots	ngsplot [35]				
	Plots	deepTools [36]				

^a^ Ideally technical replicates rather than biological replicates. Numbers in parentheses denote the samples in total. ^b^ For somatic mutation calling, a matched normal DNA sample is highly recommended. Use of “tumor-only mode” is useful only for specific purposes. ^c^ Not directly applicable to “Diff-Exp” pipeline, it instead refers to the samples from the target jobs where this pipeline starts from. Seq: Sequencing, BWA: Burrows-Wheeler Aligner, GATK: GenomeAnalysisToolkit, ANNOVAR: Annotate Variation, STAR: Spliced Transcripts Alignment to a Reference, GSEA: Gene Set Enrichment Analysis, AEI: Alu Editing Index, 4C: Chromosome Conformation Capture-on-Chip, ChIP: Chromatin Immunoprecipitation, RIP: RNA Immunoprecipitation, MACS: Model-based Analysis of ChIP-Seq, icSHAPE: in vivo click Selective 2-Hydroxyl Acylation and Profiling Experiment, MATS: Multivariate Analysis of Transcript Splicing, eCLIP: enhanced Crosslinking and Immunoprecipitation, DMR: Differentially Methylated Regions, NA: Not Applicable. Usage of the tools and packages in CSI NGS Portal and website links to their original sources are given in Supplementary Table S1. The detailed descriptions, expected input and output of the pipelines are given in Supplementary Data and on the website Docs page.Overall runtime is the approximate time elapsed for one sample to finish all the analysis steps once the job starts running, and may vary depending on the data size, pipeline parameters and server load. However, runtime for additional samples under the same job do not multiply proportionally due to the parallelisation. In case of multiple samples, all the samples start off running as soon as there are available resources on the server and keep running in parallel until they all finish. This provides an efficient means of utilising system resources, while providing results to the user as quickly as possible.

**Table 2:** Features of CSI NGS Portal.

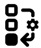	**Full-automation**	All the pipelines run from input to output without intervention with minimal user input.
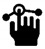	**Usability**	User-friendly and simple design with interactive tables having search, filter, sort, edit, export and share options.
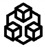	**Modularity**	Repertoire of pipelines is easy to expand complying with the existing website framework.
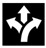	**Flexibility**	Pipelines written in virtually any script language can be integrated independently of the website code.
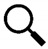	**Transparency**	The pipelines documentation are available online with the descriptions and the code.
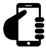	**Responsive design**	The website can be functionally displayed on multiple devices and platforms with different window/screen sizes.
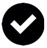	**Quality control**	FastQC report is auto-generated upon file upload, sequence and quality trimming are optionally available with multiple options.
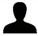	**User privacy**	No personal information is collected, secure, random cookies for authorisation and dynamic usernames for data sharing are used.
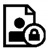	**Data privacy**	Data can be edited, deleted or shared only by the owner, expired data are completely removed from the server.
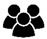	**Data sharing**	Uploaded raw FASTQ files are private to the user, results can be optionally shared/unshared with other users any time.
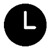	**Data availability**	Data is fully accessible via the portal until expiry (10 days, subject to revision upon usage and server capacity).
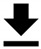	**Data download**	All the data can be downloaded to local computer with a few clicks via browser and command line.
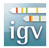	**IGV-integrated**	Alignment (.bam, .bigwig) and mutation (.vcf) data can be viewed in local IGV without downloading the original files.
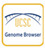	**UCSC-integrated**	Peak regions (.bigbed, .bigwig) and sites from supported pipelines can be viewed in UCSC Genome Browser online as a track hub.
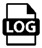	**Real-time logging**	Real-time overall job progress log and individual tool log files are generated useful for tracking and debugging.
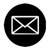	**E-mail notification**	User is notified upon job completion if e-mail address is provided during job submission (optional).
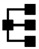	**Parallelisation**	Jobs are parallelised by multi-threading and by simultaneous run of multiple samples wherever possible.
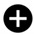	**New pipelines**	Popular and established bioinformatics pipelines for new data types are continuously added.
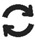	**Up-to-date**	All the tools and packages are regularly updated to the latest stable versions available.

Icons are made by Freepik and obtained from www.flaticon.com.

**Table 3:** Comparison of CSI NGS Portal to other NGS data analysis platforms.

Platform Name	Number of Pipelines/ NGS Data Types	Full Pipelines	Data Visualisation	Data Sharing	Custom Workflow Building	Code Availability	Local Installation	Registration/Login
**CSI NGS Portal**	16 ^a^	Yes	Static ^f^	Yes	No	Pipeline level	In progress	Not required
**Galaxy**	Multiple ^b^	No	Dynamic	Yes	Yes	Source level	Yes	Required
**Maser**	7 ^c^	Yes	Dynamic	Yes	Limited	No	No	Required
**RAP**	1 ^d^	Yes	Static	No	No	No	No	Required
**miRMaster**	1 ^e^	Yes	Static	No	No	No	No	Not required

Features common to all platforms such as user data upload, results download, job log, pipelines documentation, etc. are omitted, and commercially available or paid platforms are excluded from the comparison. ^a^ DNA-Seq, RNA-Seq, Diff-Exp, Pathway-Enrichment, RNA-Editing, smallRNA, 4C-Seq, ChIP-Seq, RIP-Seq, SHAPE-Seq, rMATS, circRNA, eCLIP-Seq, Bisulfite-Seq, scRNA-Seq, ngsplot-deepTools; ^b^ Pipelines are available as workflows for different data types; ^c^ RNA-Seq, ChIP-Seq, Bisulfite-Seq, Exome-Seq, De novo genome sequencing, Metagenome, CAGE/SAGE-Seq; ^d^ RNA-Seq; ^e^ miRNA-Seq; ^f^ Diverse plots in pdf format, comprehensive interactive html reports, links to IGV and UCSC track hubs are provided depending on the pipeline and accessible via single clicks on the browser.

## Supplementary Materials

The following are available online at https://www.mdpi.com/1422-0067/21/11/3828/s1, Table S1: List of tools and packages and their usage in CSI NGS Portal; Supplementary Data: Bioinformatics pipelines implemented on CSI NGS Portal.
