# Supplementary material for: CSI NGS Portal: An Online Platform for Automated NGS Data Analysis and Sharing
Source: Int J Mol Sci. 2020 May 28;21(11):3828. doi: 10.3390/ijms21113828 (PMC7312552; doi:10.3390/ijms21113828)
Supplement: Supplementary file 1 [file ijms-21-03828-s001.zip › Supplementary_Data.docx]

**Bioinformatics Pipelines Implemented on CSI NGS Portal**

CSI NGS Portal currently has 16 bioinformatics pipelines implemented, covering 11 different types of NGS data from DNA, RNA, smallRNA, 4C, ChIP, RIP, SHAPE, circRNA, eCLIP, Bisulfite-treated DNA and single cell RNA sequencing libraries.

As a rule of thumb, all the pipelines on the portal start from the FASTQ file and perform genome/transcriptome alignment generating .bam and .bigwig files. This ensures standardising the data processing with a suitable mapper for the specific task and refrain user from the tedious alignment step. Importantly, all the tools and packages used in the pipelines are regularly updated to the latest stable versions available by using a Conda (<https://conda.io/en/latest/>) environment.

1. **DNA-Seq**

This pipeline identifies and annotates somatic mutations (single nucleotide variations and indels) in the DNA of tumour samples. Both whole genome (WGS) and whole exome (WES) sequencing data can be used as the input. Use of matched normal sample is highly recommended to increase the confidence to call the somatic events although this is not theoretically required. If matched normal sample is provided, filtering of the germline mutations present in the normal sample will be performed. Otherwise “tumour-only mode” is used which may include many false positives and should be used with caution. Tumor-only mode is useful only for specific purposes, which and further details about the somatic mutation calling pipeline are described on the GATK ^1^ website (<https://software.broadinstitute.org/gatk/documentation/article?id=11136>). Currently utilisation of Panel of Normals (PoN) is not supported. After mutation calling by Mutect2 ^2^, a comprehensive annotation of the mutations is performed by ANNOVAR ^3^ including its genomic location, predicted functional impact of the mutation, implication as a clinically-associated mutation, presence in copy number variations from healthy individuals, availability in public databases such as 1000 Genomes ^4^ and COSMIC ^5^, and so on. Note that most of the functional annotation scores are available only for variants in the coding region. The output is raw and annotated somatic mutations in both .txt and .vcf formats.

1. **RNA-Seq**

This pipeline performs gene and isoform expression quantification from RNA-Seq data, as well as comparison of alternative splicing events across samples in a pairwise manner. In the case of strand-specific RNA-Seq data, all the analyses are done on both strands separately by using the strand information and the output data are provided for both forward and reverse strands (sense and antisense for expression). For gene expression, raw read counts are provided by HTSeq-count ^6^, which is the input for many downstream analyses such as differential gene expression (DESeq2 ^7^, EdgeR ^8^, etc.) and raw read counts are not directly comparable between different samples alone. A separate pipeline for differential gene/isoform expression analysis with DESeq2 ^7^ / DEXSeq ^9^ as well as gene set enrichment analysis with GSEA ^10^ is also available named as “Diff-Exp” (described next), which starts from the output of RNA-Seq job. For the isoform expression, both read counts and Transcripts Per Million (TPM) quantified by Salmon ^11^ are provided in a strand-specific manner (if available), and TPM values allow comparison between different samples. Significant alternative splicing events are provided in the “SPLICING” folder as pairwise comparison across all the samples submitted under the same job for 5 different types as described on the “Docs” page of the website. In addition, merged tables are provided in matrix format for gene (counts) and isoform (TPM) expression for all the samples.

1. **Diff-Exp**

This pipeline performs differential gene expression analysis by using DESeq2 ^7^ / DEXSeq ^9^ starting from raw read counts / exon counts using the output of an RNA-Seq job. Therefore, it is required to run an RNA-Seq job first on the batch of samples, which will automatically be available to the Diff-Exp pipeline once finished. The differentially expressed genes/isoforms are identified by comparing two groups of samples specified by the user and the samples under the same group are collapsed as technical replicates. Note that replicates are required for the estimation of dispersion, as treating single samples as replicates is no longer supported by DESeq2. The group assignment of the samples is imported from the “Diff-Exp Group” column on the “Annotate” page, and can be changed to submit a new job for a different comparison of interest. For the gene expression analysis, normalization, PCA and clustering analyses are still performed for all the samples together under the same RNA-Seq job, even though a subset of samples are used for comparison (“contrast” parameter in DESeq2). Therefore for accurate results, all RNA-Seq samples under the same job should ideally come from the same library/batch. Note that for strand-specific RNA-Seq, read counts only from the sense strand are used, therefore it's important to specify the strand specificity correctly when submitting the RNA-Seq job. Also note that to decrease the bias for lowly/not expressed genes, only genes expressed above a certain threshold are retained for differential expression analysis (those with total read counts more than 2 times of the number of samples), hence the number of genes in the output table may vary from one batch to another.

Once the differentially expressed genes are identified, pathway enrichment analysis by Reactome ^12^ is performed on the up- and down-regulated genes separately, which is described below in more detail. In addition, gene set enrichment analysis (GSEA) ^10^ is optionally available under this pipeline. Gene sets from the Molecular Signatures Database ^13^ (MSigDB) can be selected as the input, including all gene sets, 8 major collections (H: hallmark ^14^ as the default) and several sub-collections. For GSEA, the normalised read counts processed by DESeq2 and filtered for lowly expressed genes are used, and the same grouping of the samples are applied as above (Group1 vs Group2 as the “phenotype” parameter). Different options for the “permute” and “metric” parameters are used depending on the sample size, i.e. if the number of samples in either group is less than 3, “log2_Ratio_of_Classes” is applied rather than the default “Signal2Noise”, similarly if the number of samples in either group is less than 7, “gene_set” is applied rather than the default “phenotype”. The analysis results are directly viewable on the browser as a comprehensive report.

Diff-Exp pipeline can also be applied to circRNA and smallRNA jobs to identify and visualise the differentially expressed circRNAs/smallRNAs, however pathway enrichment and GSEA steps are not available for these jobs.

1. **Pathway-Enrichment**

This pipeline performs standalone pathway enrichment analysis based on Reactome ^15^ starting from a list of input gene ids (Entrez Gene ID ^16^ and/or HUGO Gene Symbol ^17^). Several plots are generated for different representations of the enriched genes and the pathways, including barplot, dotplot, cnetplot, upsetplot, heatplot, emapplot and pmcplot available in the enrichplot ^12, 18^package. This pipeline is also available as part of the “Diff-Exp” pipeline described above, where the input genes are the differentially expressed genes identified in the RNA-Seq samples provided by the user.

1. **RNA-Editing**

This pipeline, adopted from a previously published method ^19^, identifies specific nucleotide changes that occur on the RNA caused by a post- and/or co-transcriptional modification known as RNA editing. In mammals, RNA editing predominantly results in A->I(G) changes due to the deamination activity of the ADAR enzymes. This pipeline runs on RNA-Seq data alone without matched genomic DNA sequence by using a set of stringent filters to exclude potential false positives, such as known single nucleotide polymorphisms (SNPs) and spurious sites. In case of cell lines, a pre-compiled list of cell line specific DNA mutations can be optionally excluded from the editing sites, which should be done in case cell line mutations for the input samples are available. The final list of candidate editing sites are reported per sample in repetitive (Alu and Non-Alu) and non-repetitive (Unique) genomic regions. In addition, merged tables are also provided in long and wide formats which allows to compare the editing sites (only A->G and C->T changes) across all the samples submitted under the same job (wide format only if n <= 30), and includes annotation by ANNOVAR ^3^ for the genomic location and the predicted functional consequence of each variant. The reported editing sites should be further filtered by the coverage and the editing frequency with a proper cut-off for the downstream analyses, for example Coverage >= 20 and Mutation_Frequency > 0.1. The visualisation of the A->G editing sites as custom tracks is provided by a UCSC track hub, where the filtered sites are colored as red (high-confidence) and other sites are shown in black. In addition, Alu Editing Index (AEI) is calculated by using RNAEditingIndexer ^20^ to infer overall RNA editing level as a single value per sample for 6 types of mismatches (A2C, A2G, A2T, C2A, C2G, C2T), where A2GEditingIndex is the signal of the editing as an indicator of ADAR activity, and C2TEditingIndex is the highest noise (in most cases).

1. **smallRNA**

This pipeline, developed in-house, quantifies the expression of the smallRNA family including miRNAs, snoRNAs, tRNAs, rRNAs and piRNAs. The annotations of the smallRNAs are obtained as follows:

1. **miRNAs**

- downloaded from miRBase v21 ^21^, which is originally in hg38, the coordinates were then converted to hg19 using UCSC LiftOver ^22^ tool in Galaxy ^23^.

1. **snoRNAs, tRNAs and rRNAs**

- downloaded from UCSC hg19 sno/miRNA, tRNA and rmsk tracks ^24^, respectively.

1. **piRNA**

- downloaded from piRNABank ^25^.

Based on the above annotations, a smallRNA reference genome was prepared to which the raw reads are mapped by using NovoAlign (<http://www.novocraft.com/products/novoalign/>). The expression of the smallRNAs are quantified based on the Concise Idiosyncratic Gapped Alignment Report (CIGAR) string in the alignment bam file by using an in-house perl script. For the miRNAs, because the hairpin and the mature miRNAs share identical sequence but are of different length, the reads are assigned according to the mapped read length. The reads which map to a sequence longer and shorter than 30bp are counted as hairpin and mature miRNA, respectively. To reduce the output file size, raw read counts only for the expressed smallRNAs are provided in the expression output file, i.e. those with 0 read count are omitted.

1. **4C-Seq**

This pipeline identifies long-range genomic interaction regions generated by 4C-Seq experiment using the R package r3Cseq ^26^. Briefly, for each sample/replicate the raw reads are aligned to the masked version of the reference genome (masked for the gap, repetitive and ambiguous sequences) for human (hg19) or mouse (mm10, mm9) species as downloaded from the R Bioconductor repository (BSgenome.Hsapiens.UCSC.hg19.masked, BSgenome.Mmusculus.UCSC.mm10.masked, BSgenome.Mmusculus.UCSC.mm9.masked). The viewpoint chromosome, the restriction enzyme (first cutter) to digest the genome, the reads count method and the primers (forward and reverse) are the required inputs from the user. The primers must be minimum 20 bases long and uniquely mapped to the reference genome on the specified viewpoint chromosome. To count the number of reads per region, in addition to the default method “Fragment” where the restriction fragments are considered, a non-overlapping window size in the range of 2-100kb can also be selected. The number of mapped reads for each fragment/window are then counted and normalised to obtain RPM (the reads per million per fragment/window) values to perform the statistical analysis. This pipeline works with or without control samples, and also with or without replicates, however, if replicates are provided, it is compulsory also to provide control samples. The output is a text file containing the interaction regions along with the statistics and the overlapping genes, and a pdf report which provides plots for the visualisation of the interactions.

1. **ChIP-Seq**

This pipeline identifies and characterises genome-wide binding sites for DNA-protein interactions. Peak regions are called by using MACS2 ^27^ with default parameters, allowing user an option to choose for “regular” or “broad” peaks. Peak annotation and motif analysis are done by HOMER ^28^. The visualisation of the data is provided by a UCSC track hub ^29^. The output is a text file containing the peak regions with statistics and gene annotations, motif enrichment analysis results and a link to the UCSC Genome Browser ^22^ to display the peaks as custom tracks.

1. **RIP-Seq**

This pipeline, developed in-house (unpublished work), identifies and characterises genome-wide binding sites for RNA-protein interactions. Reads from the RIP-Seq sample and its control are mapped against specified reference genome by STAR ^30^ with GENCODE ^31^ transcriptome annotation. The resulting alignments are separated into two parts: (1) Exonic part consisting of alignments belonging to GENCODE annotated transcripts. (2) Non-exonic part consisting of the other alignments. Based on the number of reads mapped to the transcriptome, the larger one of the RIP and the control is shrunk down linearly to fit the size of the smaller one, thereby producing the normalised read count. The read coverage of each position is estimated as the average of normalised read counts within surrounding 150 bases. Based on the comparison of the read coverage between the IP and the control, sites with ≥ 2-fold enrichment and Poisson distribution p-value ≤ 10-5 are defined as peaks. Each peak is extended to surrounding areas until the fold enrichment dropped below 2 (note: a peak from the exonic part could span across multiple exons). Overlapping peaks are merged, and those ≤ 300bp in size are ignored. The summit of a peak is defined as the position of highest fold enrichment in the peak. This pipeline accepts only paired-end reads as the input. For each job, 1 experiment up to 10 replicates may be submitted, and each experiment targeting a different protein can be submitted as a different job. The output is text files containing the peak regions in exonic and non-exonic regions and a link to the UCSC track hub ^29^ to visualise the peaks.

1. **SHAPE-Seq**

This pipeline provides secondary structure information on RNA based on experimental constraints. The analysis is performed by using icSHAPE pipeline ^32^ and RNAfold ^33^ from the ViennaRNA package ^34^ with the default parameters unless otherwise selected. Briefly, after trimming for adapter sequences and removal of PCR duplicates, the reads are mapped to the selected human transcriptome by using bowtie2 ^35^. The transcript abundance is estimated by using Reads Per Kilobase of transcript per Million (RPKM) values and reverse transcription (RT) stops are calculated in each transcript. The background and the target RT stops from the control (DMSO) and the treated (NAI) samples, respectively, are normalised to calculate the enrichment reactivity scores for all the transcripts. These enrichment scores are further filtered to select the candidate transcripts with valid scores as well as high hit coverage and base density, where the enrichment threshold can be set by the user. The secondary structures of the substrates are then predicted with the SHAPE ^36^ reactivity scores as constraints to guide the structure prediction. The output is text files containing the reactivity scores before and after filtering, and pdf files depicting the secondary structures of the target RNAs.

1. **rMATS**

This pipeline identifies differential alternative splicing events from RNA-Seq data between test and control samples by using replicate Multivariate Analysis of Transcript Splicing (rMATS) ^37^ (<http://rnaseq-mats.sourceforge.net/>), which requires replicates as per its design. The identified events are categorised as skipped exon (SE), alternative 5’ splice site (A5SS), alternative 3’ splice site, mutually exclusive exons (MXE) and retained intron (RI). This pipeline requires replicates as the input, and the output is text files for each category. This is a standalone tool for splicing analysis in addition to the in-house developed tool available under the RNA-Seq pipeline.

1. **circRNA**

This pipeline, developed in-house, identifies circRNAs based on chimeric junction reads from STAR ^30^ alignment and quantifies their expression as read counts. Both RNA-Seq and circRNA-Seq data can be used as the input. However, circRNA enriched libraries are strongly suggested for easier detection, i.e. polyA(-), rRNA-depleted, RNase R treated for linear RNA digestion etc. PolyA selected RNA-Seq data are not useful for circRNA detection, as circRNAs do not possess polyA tails. At the end of the pipeline, circRNAs identified from all the samples under the same job are merged into one file to make comparison and filtering easy.

1. **eCLIP-Seq**

This pipeline identifies genomic locations of RNA-bound proteins. The peaks are identified by eCLIP ^38, 39^ pipeline. The output contains input-normalized peak regions for each sample. In addition, peak annotation and motif analyses are done by HOMER for highly significant peaks: -log10(pValue)>2 and log2(foldChange)>3. The visualisation of the peaks as custom tracks is provided by a UCSC Track Hub, where the peaks are colored based on their significance level (red: highly significant, blue: significant, black: non-significant).

1. **Bisulfite-Seq**

This pipeline identifies methylation pattern on bisulfite-treated genomic DNA. The leading bases and the adaptor sequences are trimmed from the reads by TrimGalore (<http://www.bioinformatics.babraham.ac.uk/projects/trim_galore/>). Then methylation calls are performed by using Bismark ^40^ with default parameters on the trimmed files, first by removing PCR duplicates by *deduplicate_bismark* script and then extracting the DNA methylation status on every cytosine site by *bismark_methylation_extractor* script. DNA methylation status are converted to bigWig format for the visualisation of the data as custom tracks by a UCSC track hub ^29^. The processing and summary reports are generated by *bismark2report* and *bismark2summary* scripts. *de-novo* differentially methylated regions (DMRs) are identified by metilene ^41^ for all pairwise sample combinations under the same job. A filtered file for significant DMRs (q < 0.05) and basic statistic plots are also provided.

1. **scRNA-Seq**

This pipeline performs single cell gene expression (scRNA-Seq) analysis for the samples generated by 10x Genomics platform by using Cell Ranger software (<https://support.10xgenomics.com/single-cell-gene-expression/software/pipelines/latest/what-is-cell-ranger>). The required inputs are read1 (R1) and read2 (R2) files in FASTQ format that are already demultiplexed. If multiple files are available for the same sample coming from different lanes, all the files can be uploaded and only 1 of them must be submitted to the pipeline, and all other files belonging to the same sample will be automatically recognized and processed together. For this to work properly, it is important that the FASTQ filenames must follow the format as described on the portal. Each sample is individually processed by *cellranger count* for feature counting, and then an aggregated analysis on all the samples under the same job is performed with *cellranger aggr*. The output is barcoded BAM, run summary, cloupe file, analysis folder, raw and filtered feature-barcode matrix files, as overviewed here. Cloupe file can be downloaded to visualise and analyse the data for finding significant genes, identifying cell types, and exploring substructure within cell clusters by following Loupe Cell Browser Tutorial. Analysis folder output and matrix files can be downloaded to directly utilize in R or other software specialized for single cell RNA-Seq data (such as Seurat, Monocle, dropEst, or others) for downstream analyses.

1. **ngsplot-deepTools**

This pipeline generates a number of useful plots to visually explore RNA-Seq and ChIP-Seq data at functional genomic regions by using both ngsplot ^42^ and deepTools ^43^ . The pipeline starts from FASTQ files and performs genome alignment as the first step using STAR ^30^ for RNA-Seq and bowtie2 ^35^ for ChIP-Seq samples. Then for each sample, genome-wide (GENOME) coverage plots are generated useful for bias detection from the bam files by ngsplot at 5 different functional genomic regions: GENEBODY, TSS, TES, EXON, CGI. If input genes are provided by the user (optional), the same plots are additionally generated limited to these genes (GENES). Note that currently only gene symbols and 1 list of genes are accepted, and genes analysis will be limited to those symbols matching NCBI RefSeq database (UCSC refGene table). In addition, OVERALL plots including all the samples under the same job are also provided by ngsplot as well as deepTools (coverage, correlation, PCA, fingerprint, heatmap, profile, gcbias) for genome-wide and for the input genes (if provided and wherever applicable).

**REFERENCES**

1. McKenna, A.; Hanna, M.; Banks, E.; Sivachenko, A.; Cibulskis, K.; Kernytsky, A.; Garimella, K.; Altshuler, D.; Gabriel, S.; Daly, M., et al., The Genome Analysis Toolkit: a MapReduce framework for analyzing next-generation DNA sequencing data. *Genome Res* **2010,** *20* (9), 1297-303.

2. Cibulskis, K.; Lawrence, M.S.; Carter, S.L.; Sivachenko, A.; Jaffe, D.; Sougnez, C.; Gabriel, S.; Meyerson, M.; Lander, E.S.; Getz, G., Sensitive detection of somatic point mutations in impure and heterogeneous cancer samples. *Nat Biotechnol* **2013,** *31* (3), 213-9.

3. Wang, K.; Li, M.; Hakonarson, H., ANNOVAR: functional annotation of genetic variants from high-throughput sequencing data. *Nucleic Acids Res* **2010,** *38* (16), e164.

4. Genomes Project, C.; Auton, A.; Brooks, L.D.; Durbin, R.M.; Garrison, E.P.; Kang, H.M.; Korbel, J.O.; Marchini, J.L.; McCarthy, S.; McVean, G.A., et al., A global reference for human genetic variation. *Nature* **2015,** *526* (7571), 68-74.

5. Tate, J.G.; Bamford, S.; Jubb, H.C.; Sondka, Z.; Beare, D.M.; Bindal, N.; Boutselakis, H.; Cole, C.G.; Creatore, C.; Dawson, E., et al., COSMIC: the Catalogue Of Somatic Mutations In Cancer. *Nucleic Acids Res* **2019,** *47* (D1), D941-D947.

6. Anders, S.; Pyl, P.T.; Huber, W., HTSeq--a Python framework to work with high-throughput sequencing data. *Bioinformatics* **2015,** *31* (2), 166-9.

7. Love, M.I.; Huber, W.; Anders, S., Moderated estimation of fold change and dispersion for RNA-seq data with DESeq2. *Genome Biol* **2014,** *15* (12), 550.

8. McCarthy, D.J.; Chen, Y.; Smyth, G.K., Differential expression analysis of multifactor RNA-Seq experiments with respect to biological variation. *Nucleic Acids Res* **2012,** *40* (10), 4288-97.

9. Anders, S.; Reyes, A.; Huber, W., Detecting differential usage of exons from RNA-seq data. *Genome Res* **2012,** *22* (10), 2008-17.

10. Subramanian, A.; Tamayo, P.; Mootha, V.K.; Mukherjee, S.; Ebert, B.L.; Gillette, M.A.; Paulovich, A.; Pomeroy, S.L.; Golub, T.R.; Lander, E.S., et al., Gene set enrichment analysis: a knowledge-based approach for interpreting genome-wide expression profiles. *Proc Natl Acad Sci U S A* **2005,** *102* (43), 15545-50.

11. Patro, R.; Duggal, G.; Love, M.I.; Irizarry, R.A.; Kingsford, C., Salmon provides fast and bias-aware quantification of transcript expression. *Nat Methods* **2017,** *14* (4), 417-419.

12. Yu, G.; He, Q.Y., ReactomePA: an R/Bioconductor package for reactome pathway analysis and visualization. *Mol Biosyst* **2016,** *12* (2), 477-9.

13. Liberzon, A.; Subramanian, A.; Pinchback, R.; Thorvaldsdottir, H.; Tamayo, P.; Mesirov, J.P., Molecular signatures database (MSigDB) 3.0. *Bioinformatics* **2011,** *27* (12), 1739-40.

14. Liberzon, A.; Birger, C.; Thorvaldsdottir, H.; Ghandi, M.; Mesirov, J.P.; Tamayo, P., The Molecular Signatures Database (MSigDB) hallmark gene set collection. *Cell Syst* **2015,** *1* (6), 417-425.

15. Fabregat, A.; Jupe, S.; Matthews, L.; Sidiropoulos, K.; Gillespie, M.; Garapati, P.; Haw, R.; Jassal, B.; Korninger, F.; May, B., et al., The Reactome Pathway Knowledgebase. *Nucleic Acids Res* **2018,** *46* (D1), D649-D655.

16. Maglott, D.; Ostell, J.; Pruitt, K.D.; Tatusova, T., Entrez Gene: gene-centered information at NCBI. *Nucleic Acids Res* **2007,** *35* (Database issue), D26-31.

17. Yates, B.; Braschi, B.; Gray, K.A.; Seal, R.L.; Tweedie, S.; Bruford, E.A., Genenames.org: the HGNC and VGNC resources in 2017. *Nucleic Acids Res* **2017,** *45* (D1), D619-D625.

18. Yu, G.; Wang, L.G.; Yan, G.R.; He, Q.Y., DOSE: an R/Bioconductor package for disease ontology semantic and enrichment analysis. *Bioinformatics* **2015,** *31* (4), 608-9.

19. Ramaswami, G.; Zhang, R.; Piskol, R.; Keegan, L.P.; Deng, P.; O'Connell, M.A.; Li, J.B., Identifying RNA editing sites using RNA sequencing data alone. *Nat Methods* **2013,** *10* (2), 128-32.

20. Roth, S.H.; Levanon, E.Y.; Eisenberg, E., Genome-wide quantification of ADAR adenosine-to-inosine RNA editing activity. *Nat Methods* **2019,** *16* (11), 1131-1138.

21. Kozomara, A.; Birgaoanu, M.; Griffiths-Jones, S., miRBase: from microRNA sequences to function. *Nucleic Acids Res* **2019,** *47* (D1), D155-D162.

22. Haeussler, M.; Zweig, A.S.; Tyner, C.; Speir, M.L.; Rosenbloom, K.R.; Raney, B.J.; Lee, C.M.; Lee, B.T.; Hinrichs, A.S.; Gonzalez, J.N., et al., The UCSC Genome Browser database: 2019 update. *Nucleic Acids Res* **2019,** *47* (D1), D853-D858.

23. Afgan, E.; Baker, D.; Batut, B.; van den Beek, M.; Bouvier, D.; Cech, M.; Chilton, J.; Clements, D.; Coraor, N.; Gruning, B.A., et al., The Galaxy platform for accessible, reproducible and collaborative biomedical analyses: 2018 update. *Nucleic Acids Res* **2018,** *46* (W1), W537-W544.

24. Karolchik, D.; Hinrichs, A.S.; Furey, T.S.; Roskin, K.M.; Sugnet, C.W.; Haussler, D.; Kent, W.J., The UCSC Table Browser data retrieval tool. *Nucleic Acids Res* **2004,** *32* (Database issue), D493-6.

25. Sai Lakshmi, S.; Agrawal, S., piRNABank: a web resource on classified and clustered Piwi-interacting RNAs. *Nucleic Acids Res* **2008,** *36* (Database issue), D173-7.

26. Thongjuea, S.; Stadhouders, R.; Grosveld, F.G.; Soler, E.; Lenhard, B., r3Cseq: an R/Bioconductor package for the discovery of long-range genomic interactions from chromosome conformation capture and next-generation sequencing data. *Nucleic Acids Res* **2013,** *41* (13), e132.

27. Zhang, Y.; Liu, T.; Meyer, C.A.; Eeckhoute, J.; Johnson, D.S.; Bernstein, B.E.; Nusbaum, C.; Myers, R.M.; Brown, M.; Li, W., et al., Model-based analysis of ChIP-Seq (MACS). *Genome Biol* **2008,** *9* (9), R137.

28. Heinz, S.; Benner, C.; Spann, N.; Bertolino, E.; Lin, Y.C.; Laslo, P.; Cheng, J.X.; Murre, C.; Singh, H.; Glass, C.K., Simple combinations of lineage-determining transcription factors prime cis-regulatory elements required for macrophage and B cell identities. *Mol Cell* **2010,** *38* (4), 576-89.

29. Raney, B.J.; Dreszer, T.R.; Barber, G.P.; Clawson, H.; Fujita, P.A.; Wang, T.; Nguyen, N.; Paten, B.; Zweig, A.S.; Karolchik, D., et al., Track data hubs enable visualization of user-defined genome-wide annotations on the UCSC Genome Browser. *Bioinformatics* **2014,** *30* (7), 1003-5.

30. Dobin, A.; Davis, C.A.; Schlesinger, F.; Drenkow, J.; Zaleski, C.; Jha, S.; Batut, P.; Chaisson, M.; Gingeras, T.R., STAR: ultrafast universal RNA-seq aligner. *Bioinformatics* **2013,** *29* (1), 15-21.

31. Frankish, A.; Diekhans, M.; Ferreira, A.M.; Johnson, R.; Jungreis, I.; Loveland, J.; Mudge, J.M.; Sisu, C.; Wright, J.; Armstrong, J., et al., GENCODE reference annotation for the human and mouse genomes. *Nucleic Acids Res* **2019,** *47* (D1), D766-D773.

32. Flynn, R.A.; Zhang, Q.C.; Spitale, R.C.; Lee, B.; Mumbach, M.R.; Chang, H.Y., Transcriptome-wide interrogation of RNA secondary structure in living cells with icSHAPE. *Nat Protoc* **2016,** *11* (2), 273-90.

33. Lorenz, R.; Hofacker, I.L.; Stadler, P.F., RNA folding with hard and soft constraints. *Algorithms Mol Biol* **2016,** *11*, 8.

34. Lorenz, R.; Bernhart, S.H.; Honer Zu Siederdissen, C.; Tafer, H.; Flamm, C.; Stadler, P.F.; Hofacker, I.L., ViennaRNA Package 2.0. *Algorithms Mol Biol* **2011,** *6*, 26.

35. Langmead, B.; Salzberg, S.L., Fast gapped-read alignment with Bowtie 2. *Nat Methods* **2012,** *9* (4), 357-9.

36. Merino, E.J.; Wilkinson, K.A.; Coughlan, J.L.; Weeks, K.M., RNA structure analysis at single nucleotide resolution by selective 2'-hydroxyl acylation and primer extension (SHAPE). *J Am Chem Soc* **2005,** *127* (12), 4223-31.

37. Shen, S.; Park, J.W.; Lu, Z.X.; Lin, L.; Henry, M.D.; Wu, Y.N.; Zhou, Q.; Xing, Y., rMATS: robust and flexible detection of differential alternative splicing from replicate RNA-Seq data. *Proc Natl Acad Sci U S A* **2014,** *111* (51), E5593-601.

38. Van Nostrand, E.L.; Pratt, G.A.; Shishkin, A.A.; Gelboin-Burkhart, C.; Fang, M.Y.; Sundararaman, B.; Blue, S.M.; Nguyen, T.B.; Surka, C.; Elkins, K., et al., Robust transcriptome-wide discovery of RNA-binding protein binding sites with enhanced CLIP (eCLIP). *Nat Methods* **2016,** *13* (6), 508-14.

39. Van Nostrand, E.L.; Nguyen, T.B.; Gelboin-Burkhart, C.; Wang, R.; Blue, S.M.; Pratt, G.A.; Louie, A.L.; Yeo, G.W., Robust, Cost-Effective Profiling of RNA Binding Protein Targets with Single-end Enhanced Crosslinking and Immunoprecipitation (seCLIP). *Methods Mol Biol* **2017,** *1648*, 177-200.

40. Krueger, F.; Andrews, S.R., Bismark: a flexible aligner and methylation caller for Bisulfite-Seq applications. *Bioinformatics* **2011,** *27* (11), 1571-2.

41. Juhling, F.; Kretzmer, H.; Bernhart, S.H.; Otto, C.; Stadler, P.F.; Hoffmann, S., metilene: fast and sensitive calling of differentially methylated regions from bisulfite sequencing data. *Genome Res* **2016,** *26* (2), 256-62.

42. Shen, L.; Shao, N.; Liu, X.; Nestler, E., ngs.plot: Quick mining and visualization of next-generation sequencing data by integrating genomic databases. *BMC Genomics* **2014,** *15*, 284.

43. Ramirez, F.; Ryan, D.P.; Gruning, B.; Bhardwaj, V.; Kilpert, F.; Richter, A.S.; Heyne, S.; Dundar, F.; Manke, T., deepTools2: a next generation web server for deep-sequencing data analysis. *Nucleic Acids Res* **2016,** *44* (W1), W160-5.
