# Supplementary material for: CSI NGS Portal: An Online Platform for Automated NGS Data Analysis and Sharing
Source: Int J Mol Sci. 2020 May 28;21(11):3828. doi: 10.3390/ijms21113828 (PMC7312552; doi:10.3390/ijms21113828)
Supplement: Supplementary file 1 [file ijms-21-03828-s001.zip › Supplementary_Table_S1.pdf]

Supplementary Table S1: List of tools & packages and their usage in CSI NGS Portal
All the tools and packages are regularly updated to the latest stable versions (unless version number is explicitly specified).
An exhaustive list of dependencies are omitted.

Tool/package	Bioinformatics Pipeline	Usage	Link
**Website Development**			
Apache2	NA	Web server	https://httpd.apache.org/
PHP	NA	Website interface and functionality	https://www.php.net/
HTML5	NA	Website interface and functionality	https://www.w3.org/TR/html5/
CSS	NA	Website styling	https://www.w3.org/Style/CSS/
Bootstrap v3.3.6	NA	Interactive tables and responsive design	https://getbootstrap.com/
JavaScript	NA	Dynamic features	https://developer.mozilla.org/en-US/docs/Web/JavaScript
jQuery v1.12.1	NA	Dynamic features	https://jquery.com/
jQuery File Upload	NA	Data upload interface	https://github.com/blueimp/jQuery-File-Upload
**Database**			
MariaDB	NA	Data storage	https://mariadb.org/
**Programming Languages**			
Bash	All	Wrapper scripts for pipeline development	https://www.gnu.org/software/bash/
Python	All	Running of packages in conda environment	https://www.python.org/ , https://anaconda.com
Perl	All	Running of in-house and external pipelines	https://www.perl.org/
R	Diff4C-Seq, Diff-Exp, Pathway-Enrichment	Running CRAN and Bioconductor packages	https://www.r-project.org/
**Anaconda2 Packages**			
FastQC	All	Quality control	https://www.bioinformatics.babraham.ac.uk/projects/fastqc/
Trimmomatic	All	Adapter removal and read trimming	http://www.usadellab.org/cms/?page=trimmomatic
bedtools	All	Genome arithmetic	https://bedtools.readthedocs.io
Samtools	All	Manipulation of HTS files	https://github.com/samtools/samtools
Picard	DNA-Seq, RIP-Seq	Manipulation of HTS files	https://github.com/broadinstitute/picard
BWA (mem)	DNA-Seq, RNA-Editing, 4C-Seq	Read mapping	https://github.com/lh3/bwa
Bowtie2	SHAPE-Seq, Bisulfite-Seq, ngsplot-deepTools	Read mapping	http://bowtie-bio.sourceforge.net/bowtie2/index.shtml
NovoAlign	smallRNA	Read mapping	http://www.novocraft.com/products/novoalign/
STAR	RNA-Seq, RIP-Seq, rMATS, circRNA. eCLIP-Seq, ngsplot-deepTools	RNA-Seq read alignment	https://github.com/alexdobin/STAR
GATK4 (Mutect2)	DNA-Seq	Genome analysis toolkit (Mutation calling)	https://software.broadinstitute.org/gatk/documentation/article?id=11136
HTSeq (count)	RNA-Seq	Gene expression quantification	https://htseq.readthedocs.io/en/master/count.html
Salmon	RNA-Seq	Isoform expression quantification	https://salmon.readthedocs.io/en/latest/salmon.html
MACS2	ChIP-Seq, RIP-Seq	Identification of transcription factor binding sites	https://github.com/taoliu/MACS
Homer	ChIP-Seq	Motif enrichment analysis	http://homer.ucsd.edu/homer/motif/
ViennaRNA (RNAfold)	SHAPE-Seq	RNA secondary structure prediction	https://www.tbi.univie.ac.at/RNA/RNAfold.1.html
TrimGalore/Cutadapt	RIP-Seq, Bisulfite-Seq	Adapter removal and read trimming	https://github.com/FelixKrueger/TrimGalore
Singularity	eCLIP-Seq	Running eCLIP container	https://www.sylabs.io/guides/2.6/user-guide/
Bismark	Bisulfite-Seq	Methylation calling of bisulfite treated sequencing reads	https://www.bioinformatics.babraham.ac.uk/projects/bismark/
metilene	Bisulfite-Seq	Identification of differentially metylated regions	https://www.bioinf.uni-leipzig.de/Software/metilene/
**R CRAN Packages**			
R ggplot2	Diff-Exp, Pathway-Enrichment	Generation of plots and data visualisation	https://cran.r-project.org/web/packages/ggplot2/index.html
R superheat	Diff-Exp	Heatmap generation	https://rlbarter.github.io/superheat/
R tidyverse	4C-Seq, Diff-Exp, Pathway-Enrichment	Data manipulation	https://www.tidyverse.org/
R RMySQL	Diff-Exp	Database connection	https://cran.r-project.org/web/packages/RMySQL/index.html
**R Bioconductor Packages**			
R Bioconductor r3Cseq	4C-Seq	Identification of 4C interactions	https://www.bioconductor.org/packages/release/bioc/html/r3Cseq.html
R Bioconductor BSgenome	4C-Seq	Reference genome annotations	https://bioconductor.org/packages/release/bioc/html/BSgenome.html
R Bioconductor org.Hs.eg.db	Diff-Exp	Reference genome annotations	https://bioconductor.org/packages/release/data/annotation/html/org.Hs.eg.db.html
R Bioconductor DESeq2	Diff-Exp	Differential gene expression analysis	https://www.bioconductor.org/packages/release/bioc/html/DESeq2.html
R Bioconductor DEXSeq	Diff-Exp	Differential exon usage analysis	https://bioconductor.org/packages/release/bioc/html/DEXSeq.html
R Bioconductor regionReport	Diff-Exp	Generation of interactive report for DESeq2 results	https://www.bioconductor.org/packages/release/bioc/html/regionReport.html
R Bioconductor biomaRt	Diff-Exp	ID mapping and conversion	https://www.bioconductor.org/packages/release/bioc/html/biomaRt.html
R Bioconductor ReactomePA	Diff-Exp, Pathway-Enrichment	Pathway enrichment analysis	https://www.bioconductor.org/packages/release/bioc/html/ReactomePA.html
R Bioconductor enrichplot	Diff-Exp, Pathway-Enrichment	Visualisation of pathway enrichment results	https://bioconductor.org/packages/release/bioc/html/enrichplot.html
R Bioconductor DOSE	Diff-Exp, Pathway-Enrichment	Visualisation of pathway enrichment results	https://bioconductor.org/packages/release/bioc/html/DOSE.html
**Standalone Software**			
ANNOVAR	DNA-Seq, RNA-Editing, eCLIP-Seq	Annotation of variants and peaks	http://annovar.openbioinformatics.org
GSEA	Diff-Exp	Gene set enrichment analysis	https://www.gsea-msigdb.org/gsea/index.jsp
icSHAPE	SHAPE-Seq	SHAPE reactivity calculation	https://github.com/qczhang/icSHAPE
rMATS	rMATS	Identification of differential alternative splicing events	http://rnaseq-mats.sourceforge.net/
eCLIP	eCLIP-Seq	Identification of genomic locations of RNA-bound proteins	https://github.com/YeoLab/eclip
Cell Ranger	scRNA-Seq	Single cell gene expression analysis	https://support.10xgenomics.com/single-cell-gene-expression/software/pipelines/latest/what-is-cell-ranger
ngsplot	ngsplot-deepTools	Visual exploration at functional genomic regions	https://github.com/shenlab-sinai/ngsplot
deepTools	ngsplot-deepTools	Visual exploration at functional genomic regions	https://github.com/deeptools/deeptools
